# Human Developmental Enhancers Conserved between Deuterostomes and Protostomes

**DOI:** 10.1371/journal.pgen.1002852

**Published:** 2012-08-02

**Authors:** Shoa L. Clarke, Julia E. VanderMeer, Aaron M. Wenger, Bruce T. Schaar, Nadav Ahituv, Gill Bejerano

**Affiliations:** 1 Department of Genetics, Stanford University, Stanford, California, United States of America; 2 Department of Bioengineering and Therapeutic Sciences, University of California San Francisco, San Francisco, California, United States of America; 3 Institute for Human Genetics, University of California San Francisco, San Francisco, California, United States of America; 4 Department of Computer Science, Stanford University, Stanford, California, United States of America; 5 Department of Developmental Biology, Stanford University, Stanford, California, United States of America; University of Münster, Germany

## Abstract

The identification of homologies, whether morphological, molecular, or genetic, is fundamental to our understanding of common biological principles. Homologies bridging the great divide between deuterostomes and protostomes have served as the basis for current models of animal evolution and development. It is now appreciated that these two clades share a common developmental toolkit consisting of conserved transcription factors and signaling pathways. These patterning genes sometimes show common expression patterns and genetic interactions, suggesting the existence of similar or even conserved regulatory apparatus. However, previous studies have found no regulatory sequence conserved between deuterostomes and protostomes. Here we describe the first such enhancers, which we call bilaterian conserved regulatory elements (Bicores). Bicores show conservation of sequence and gene synteny. Sequence conservation of Bicores reflects conserved patterns of transcription factor binding sites. We predict that Bicores act as response elements to signaling pathways, and we show that Bicores are developmental enhancers that drive expression of transcriptional repressors in the vertebrate central nervous system. Although the small number of identified Bicores suggests extensive rewiring of cis-regulation between the protostome and deuterostome clades, additional Bicores may be revealed as our understanding of cis-regulatory logic and sample of bilaterian genomes continue to grow.

## Author Summary

Flies and worms have long served as valuable model organisms for the study of human development and health. Despite the great morphological and evolutionary distance between them, humans, flies, and worms share many commonalities. Each develops from three major germ layers and is patterned along the two major spatial axes. At the molecular level, development in these widely diverged species is often controlled by the same signaling pathways activating members of the same transcription factor and target gene families, shared since the common ancestor of humans, flies, and worms. And yet, at the gene regulatory level, humans and flies or worms seem starkly different, with not a single regulatory region shared across the phyla. Here we discover the first two examples of developmental enhancers conserved between deuterostomes (ranging from human to sea urchins) and protostomes (a large clade that includes flies and worms). We show evidence that these ancient regulatory loci retain the capacity to respond to the same signaling pathways in these widely diverged organisms, and we show that they have been co-opted, along with the molecular pathways that control them, to pattern the vertebrate nervous systems. Our screen supports large scale regulatory rewiring, while offering the first intriguing outliers.

## Introduction

The bilaterian tree unites two major clades, deuterostomes (e.g. humans) and protostomes (e.g. flies) [1]. Protostome species such as insects, nematodes, annelids, and mollusks have served as invaluable model organisms. Much of the utility of these model systems stems from fundamental homologies between the two clades. Across bilaterians, early embryos undergo gastrulation to form three germ layers. These germ layers are patterned along dorsal-ventral and anterior-posterior axes. Underlying these processes are ancient conserved signaling pathways and transcription factors, often interacting as part of conserved genetic circuits. In both deuterostomes and protostomes, the precise expression of each circuit component depends on *cis*-regulatory elements [2], [3]. *Cis*-regulatory elements are genomic regions that transcription factors bind in order to modify the expression of a target gene [4].

*Cis*-regulatory elements are often identified as conserved non-coding elements (CNEs) [5]–[8]. Among closely related species, CNEs can show extreme conservation. For example, the human genome contains hundreds of non-coding ultraconserved elements that align to mouse and rat with 100 percent identity across 200 bases or more [5]. Many of these elements function as developmental enhancers [7]. Protostome genomes contain a distinct set of similarly ultraconserved elements [9]. Strikingly, in contrast to the genes they regulate, no *cis*-regulatory elements have previously been found to be conserved between deuterostomes and protostomes [5], [6], [10]–[12] (see Text S1). Even the oldest known enhancer, conserved between deuterostomes and the cnidarian sea anemone, has not been found to be conserved in protostomes [12]. These observations may suggest that the *cis*-regulatory component of genetic circuits has been completely rewired between deuterostomes and protostomes. Alternatively, it may be that some ancestral regulatory regions are conserved between these clades and have remained elusive due to limitations in our tools and our sample of bilaterian genomes. If conserved *cis*-regulatory elements do exist, such elements offer a new avenue for exploring how developmental logic is encoded in the genome and how this logic evolves.

Here we present the first examples of *cis*-regulatory elements conserved between deuterostomes and protostomes. These elements have conserved sequence and gene synteny. The conserved sequence reflects conservation of a series of transcription factor binding sites, and we show that these elements function as developmental enhancers.

## Results/Discussion

### Discovery of bilaterian conserved regulatory elements

**Figure 1:**
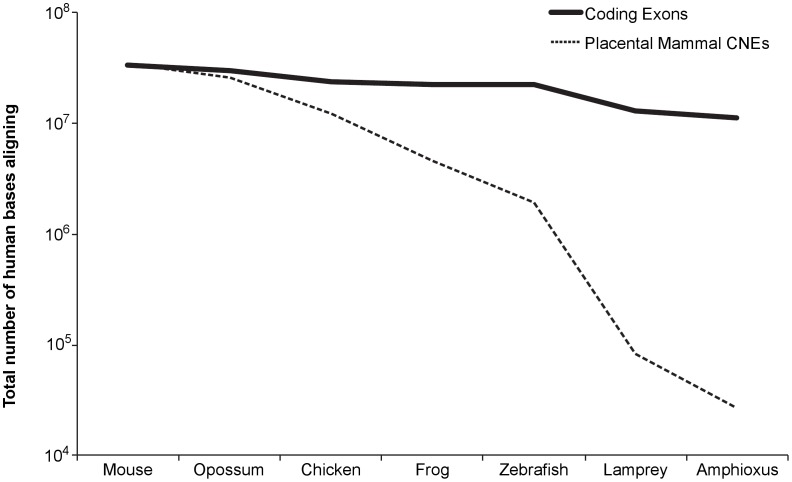
Total number of bases (coding versus conserved non-coding) in human that align to each species. Species are ordered at progressively greater evolutionary distances. Placental mammal CNEs – placental mammal conserved non-coding elements.

Conservation of non-coding sequence is rare compared to coding sequence, even over much shorter evolutionary distances than that between deuterostomes and protostomes. For example, nearly a third of human coding bases (11 Mb out of 34 Mb) align to the amphioxus genome. In stark contrast, less than one percent of CNE bases (<0.3 Mb out of 34 Mb) align to amphioxus (Figure 1).

To screen for enhancers conserved between deuterostome and protostome species, we first defined a set of vertebrate conserved non-coding elements (vertCNEs) which are human non-coding regions well-conserved in at least a subset of vertebrates (see methods). In total, we constructed a conservative set of 8,069 vertCNEs. We were especially stringent in filtering out sequences with evidence for potential coding functions (exonic or other functional RNA). vertCNEs show exceptional enrichment in GREAT [13] for clustering near transcription factors and developmental genes (Table S1). We used lastz [14] to query these elements against three previously published non-vertebrate deuterostome genomes – ciona [15], amphioxus [16], and sea urchin [17] (Figure S1). We filtered hits for both quality of alignment and for conserved gene synteny. We thus found five candidate elements showing conservation between vertebrates and at least one invertebrate (Table S2). In order to comprehensively define the extent of conservation of these elements, we searched each against all publicly available sequence data for non-vertebrate metazoans. This search included genome assemblies for 47 species (Table S3) (∼16 Gb) and NCBI trace data for 134 species (∼219 Gb). We also searched all Genbank sequence data, which included 530 species with at least 100 kilobases of sequence (∼21 Gb). Of the five candidates, two elements stood out as having conservation of sequence and gene synteny in protostome species. We call these elements bilaterian conserved regulatory elements (Bicores).

**Figure 2:**
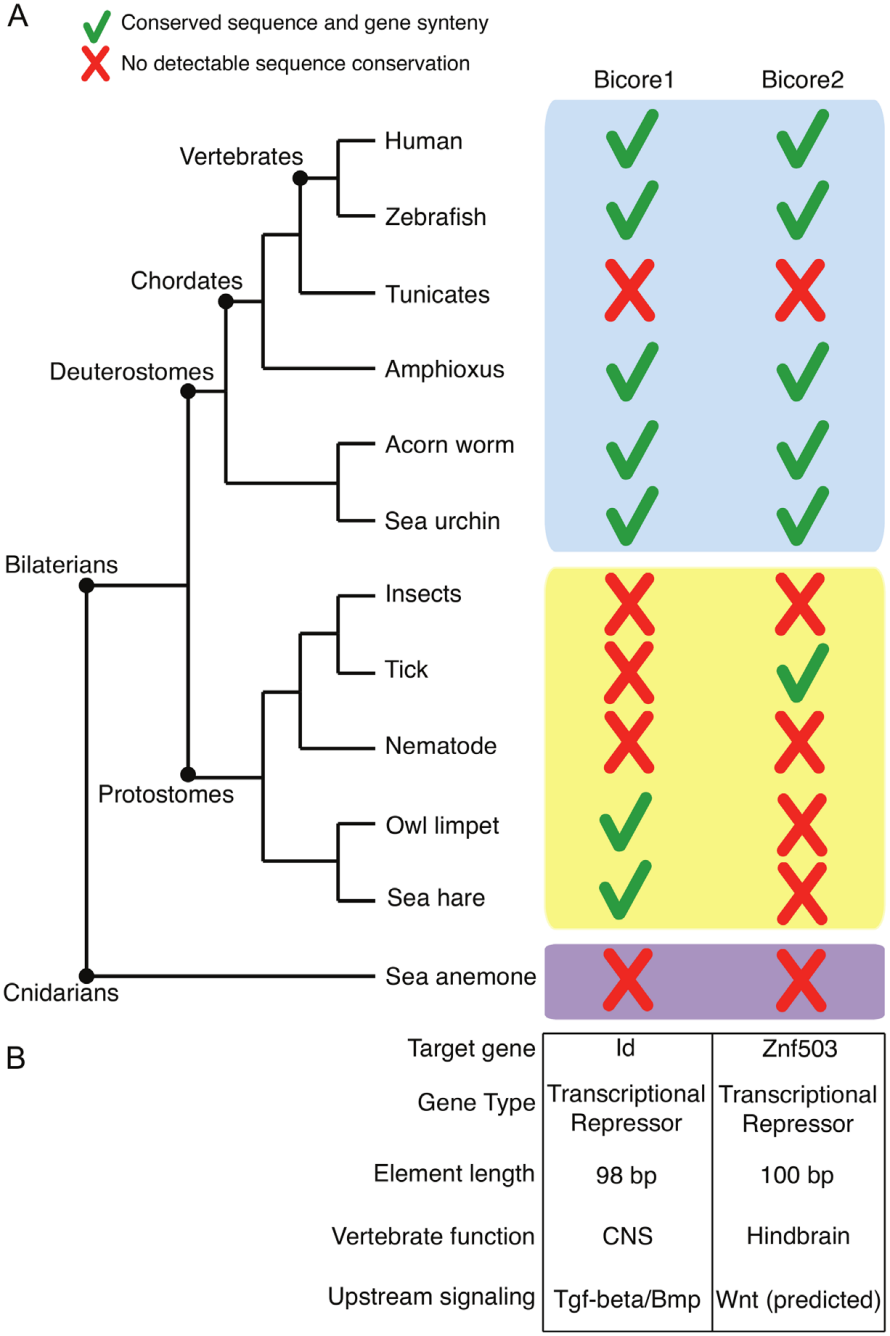
Ancient enhancers. (A) Pattern of conservation of Bicores across the metazoan tree. Green checks denote conservation of sequence and gene synteny. Red crosses denote no detectable sequence conservation. (B) Characteristics of Bicores. CNS – central nervous system.

Bicore1 is found in two protostome species - *Aplysia californica* (sea hare) and *Lottia gigantea* (owl limpet). Bicore2 is found in the protostome *Ixodes scapularis* (tick) (Figure 2). Together, Bicore1 and Bicore2 are the first examples of human CNEs conserved between deuterostome and protostome species.

### Bicore1

**Figure 3:**
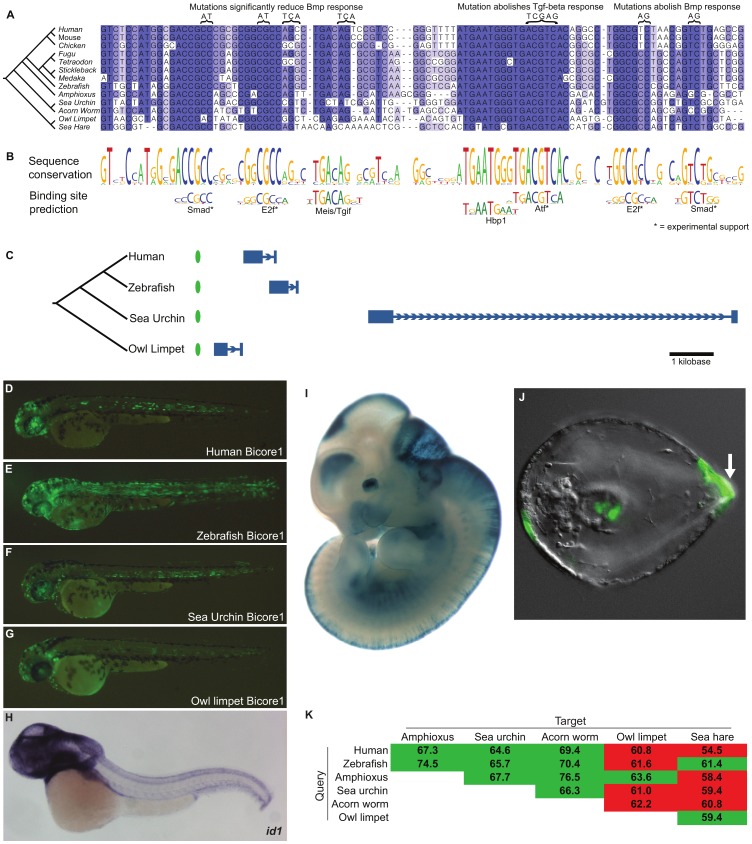
Bicore1 is a bilaterian conserved enhancer. (A) Multiple alignment of Bicore1. Above the alignment are mutations that reduce the *cis*-regulatory function of Bicore1 in response to Tgf-beta/Bmp signaling in a luciferase assay [20], [21], [24]. (B) Conservation profile of Bicore1 showing that conserved blocks in the alignment correspond to transcription factor binding preferences. (C) Each instance of Bicore1 (green oval) is syntenic to a conserved *Id* gene (blue gene structure). (D–G) Zebrafish transgenic assay showing (D) human, (E) zebrafish, (F) sea urchin, (G) owl limpet instances of Bicore1 drive expression in the central nervous system at 48 hours. (H) Whole-mount *in situ* hybridization of *id1* in zebrafish shows expression throughout the central nervous system, courtesy of zfin.org. (I) A human genomic region containing Bicore1 drives expression in the forebrain, midbrain, hindbrain, neural tube, and eye of embryonic day 11.5 mice, courtesy of enhancer.lbl.gov. (J) A sea urchin genomic region drives expression in the aboral ectoderm (arrow) in the early pluteus stage sea urchin larva. (K) Pairwise percent identities of Bicore1 sequences. Green cells indicate a query sequence (row) that detected Bicore1 in the target (column). Red cells indicate query sequences that did not detect Bicore1 in the target.

Each instance of Bicore1 is conserved upstream of a conserved *Id* (Inhibitor of DNA binding) ortholog (Figure 3C; Table S4). Id genes encode helix-loop-helix proteins that bind bHLH transcription factors, acting as transcriptional repressors. Id factors are known to inhibit cells from terminally differentiating, promoting progenitor states [18]. Mammalian genomes contain four Id genes. Bicore1 occurs upstream of *Id1*. In addition, we found mammalian paralogs of Bicore1 upstream of *Id2* and *Id4* (Figure S2; Table S4).

A multiple alignment of Bicore1 shows a striking pattern of sequence conservation (Figure 3A–3B). Short stretches of 5–10 base pairs are highly conserved, separated by stretches of non-conserved sequence. The highly conserved 5–10mers have allowed few substitutions and have completely resisted indels across species. These short conserved sequences match closely to the known binding preference of transcription factors (Figure 3B).

Two conserved sites match the primary and secondary binding preference of Smad transcription factors. Smad factors act downstream of Tgf-beta/Bmp signaling [19], suggesting that Bicore1 may be responsive to this pathway. In fact, previous studies have shown that, in a mammalian cell line, these conserved Smad sites are bound by smad transcription factors in response to Tgf-beta/Bmp signaling. These studies further showed that a region containing Bicore1 drives expression of a luciferase reporter in response to Bmp. Mutation of the Smad sites reduces or abolishes this Bmp response [20], [21]. We also see conserved matches to the E2f binding motif. E2f factors are known to act as Smad cofactors in response to Tgf-beta [22]. Mutations of these conserved E2f sites reduce Bmp responsiveness in a luciferase assay [20], [21]. ChIP-seq data also supports binding of E2f factors to this region [23]. The most highly conserved region of Bicore1 corresponds to an 8 base pair palindromic sequence that is perfectly conserved across clades. This sequence matches the binding preference of Atf factors. In mammalian cells, Atf forms a complex with Smad and directly binds Bicore1 [24]. This binding event occurs in response to Tgf-beta signaling and leads to the repression of a luciferase reporter. Mutation of the Atf site abolishes the ability of Bicore1 to repress luciferase expression in response to Tgf-beta [24]. Altogether, cell line experiments show that Bicore1 is a Tgf-beta/Bmp responsive *cis*-regulatory element; Smad, E2f, and Atf factors bind the element, and the conserved Smad, E2f, and Atf sites are necessary for its function. Thus, Bicore1 has conserved a series of transcription factor binding sites that have maintained order, orientation, and spacing for over more than 600 million years [25] of evolution.

To test if instances of Bicore1 have a conserved capacity to function as an enhancer *in vivo*, we used a zebrafish transient transgenic enhancer assay. We tested the human, zebrafish, sea urchin, and owl limpet Bicore1 sequences. These short ∼100 base pair sequences are fairly diverged from one another, with all but the human-zebrafish pairwise similarity at 60–65 percent identity. At 21 hours post-fertilization (hpf), the constructs drove strong expression throughout the embryo. Zebrafish *id1* expression at this time point, measured by whole-mount *in situ* hybridization (WISH), is similar (Figure S3). At 48 hours, we found that all four constructs drove scattered expression throughout the central nervous system (CNS) (Figure 3D–3G and Table S6), congruent with *id1* expression at this time [26] (Figure 3H). In addition to CNS, we saw expression in the notochord, a structure in which previous WISH experiments have not detected *id1* expression. It is possible that Bicore1 enhances the weak notochord background of our expression vector (see methods; Figure S4).

Further support for Bicore1 functioning as a CNS enhancer in vertebrates is provided by mouse experiments. A human construct containing Bicore1 was previously tested in a mouse transgenic enhancer assay [8]. At embryonic day 11.5, this construct drives expression in the forebrain, midbrain, hindbrain, neural tube, and eye (Figure 3I), closely matching *Id1* expression in mouse [27].

Among the protostome species that have conserved Bicore1 (owl limpet and sea hare), transgenic enhancer assays are not yet well developed. However, such assays have been described in sea urchin [28]. We tested a construct containing the sea urchin Bicore1 in a transgenic sea urchin assay. The construct drove expression in the aboral ectoderm of developing sea urchin embryos (75% of embryos) (Figure 3J). Whole-mount *in situ* hybridization experiments in a closely related sea urchin species have shown that *id* is expressed specifically in the aboral ectoderm during sea urchin development. Moreover, overexpressing Bmp expands the *id* expression pattern, and blocking Bmp signaling greatly diminishes *id* expression [29]. These data provide evidence that Bicore1 functions as a developmental enhancer of *id* in sea urchins.

### Bicore2

**Figure 4:**
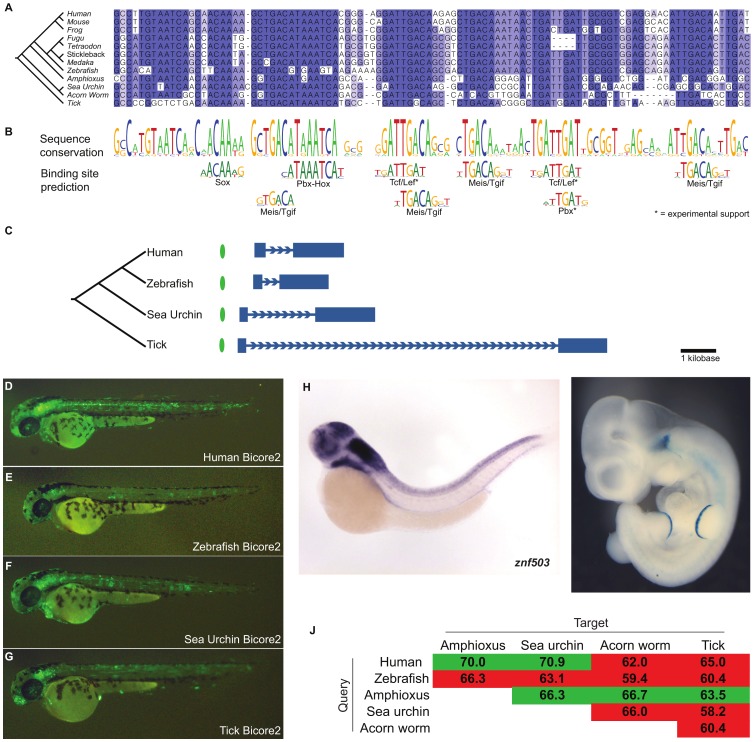
Bicore2 is a bilaterian conserved enhancer. (A) Multiple alignment of Bicore2. (B) Conservation profile of Bicore2 showing that conserved blocks in the alignment correspond to transcription factor binding preferences. (C) Each instance of Bicore2 (green oval) is syntenic to a conserved instance of *Znf503* (blue gene structure). (D–G) Zebfrafish transgenic enhancer assay showing (D) human, (E) zebrafish, (F) sea urchin, (G) tick instances of Bicore2 drive expression in the hindbrain at 48 hours. (H) Whole-mount *in situ* hybridization of Znf503 shows expression in the hindbrain, courtesy of zfin.org. (I) A human region containing Bicore2 drives expression in the hindbrain and the apical ectoderm of the limb in embryonic day 11.5 mice. (J) Pairwise percent identities of Bicore2 sequences. Green cells indicate a query sequence (row) that detected Bicore2 in the target (column). Red cells indicate query sequences that did not detect Bicore2 in the target.

Bicore2 is conserved upstream of *Znf503* (Figure 4C; Table S5), a gene which encodes a zinc-finger transcription factor predicted to act as a transcriptional repressor in deuterostomes and protostomes [30], [31]. Znf503 functions as a regulator of vertebrate hindbrain development [32], [33]. Transcription factor binding site analysis shows that Bicore2 has conserved several sequences matching the Meis/Tgif family binding preference as well as sequences matching Hox and Pbx binding preferences (Figure 4B). Among vertebrates, these factors are known to form a complex that functions during hindbrain development [34], [35]. These factors are also known to interact in protostome species [36], [37]. Bicore2 also contains highly conserved Tcf/Lef family binding sites that are supported by ChIP-seq data in mammalian cell lines [23]. Tcf/Lef transcription factors act downstream of Wnt signaling. In both deuterostomes and protostomes, Wnt signaling defines the anterior-posterior axis during development [38]. In vertebrates, Wnt signaling has also been shown to be necessary in defining the midbrain-hindbrain boundary [39].

We examined human, zebrafish, sea urchin, and tick versions of Bicore2 in a zebrafish transient transgenic enhancer assay. All four constructs drove consistent expression in the hindbrain (Figure 4D–4G), recapitulating the zebrafish *znf503* expression pattern [26] (Figure 4H). Interestingly, the zebrafish Bicore2 stood out as having the weakest expression. Zebrafish also stands out in the multiple alignment as having mutated three highly conserved bases, interrupting a predicted Meis-Pbx-Hox binding site (Figure 4A–4B). Two other sequenced fish, tetraodon and fugu, have deleted a highly conserved site predicted to bind Tcf/lef and Pbx. It is possible that in some fish, Bicore2 function has been modulated.

We also tested a human construct containing Bicore2 in a mouse assay. The construct drives expression in the hindbrain (5/6 embryos) and apical ectoderm of the limb (4/6 embryos) at embryonic day 11.5 (Figure 4I). This pattern of hindbrain and limb expression matches previously reported *Znf503* expression in mouse [40].

### Perspectives

In this study, we have identified the first examples of *cis*-regulatory sequence conserved between deuterostomes and protostomes. These bilaterian conserved regulatory elements (Bicores) are developmental enhancers that encode complex patterns of transcription factor binding sites. Bicore1 is an enhancer of Id. Binding site analysis predicts that it functions as a Bmp responsive element, and several lines of experimental evidence support this prediction. In vertebrates, Bicore1 drives expression in the developing central nervous system. In sea urchin, Bicore1 drives expression in the aboral ectoderm, a structure that goes on to form the squamous epithelium of the late larval wall [41]. Although the vertebrate nervous system and the urchin aboral ectoderm are unlikely to be homologous structures, it is reasonable to hypothesize that they might utilize similar genetic circuits. Both are ectodermal structures that define analogous axes (dorsal-ventral and aboral-oral) through Bmp signaling.

Further work will be needed to determine how Bicore1 functions in those protostome species that have conserved the sequence. We suspect that protostomes like owl limpet and sea hare also use Bicore1 as a Bmp responsive enhancer to drive *id* expression in ectodermal structures. As enhancer assays are developed in these species, we can begin testing this hypothesis. It is interesting to note that in *Drosophila melanogaster*, Emc (ortholog to Id) is not expressed in the ectoderm during development [42]. Further, constitutively active Dpp (ortholog to Bmp) signaling does not alter Emc expression [43]. Loss of Bicore1 in drosophila is consistent with these observations.

Bicore2 is an enhancer of *Znf503*. We predict from binding site analysis that it acts as a response element to Wnt signaling. In vertebrate embryos, Bicore2 drives expression in the hindbrain, an ectoderm derived structure. It may seem surprising that CNS enhancers are conserved in species that lack central nervous systems. In fact, we can infer that Bicores existed in the urbilaterian ancestor, long before the process of neurulation and the vertebrate central nervous system ever emerged. It is well established that as the vertebrate nervous system evolved, it took advantage of preexisting transcription factors and signaling pathways [44], [45]. We can now appreciate that in addition to these ancient genes, ancient *cis*-regulatory integrators, in the form of rigid enhancers, were also coopted into vertebrate nervous system development. In fact, it is expected that when key regulatory genes and pathways are activated in a new context, they initially affect downstream targets via pre-existing cis-regulatory regions.

Several past studies have searched for *cis*-regulatory elements conserved between deuterostomes and protostomes (see Text S1). However, these studies focused on searching the genomes of the most commonly used protostome model organisms, drosophila and caenorhabditis. These model organisms correspond to two of the three lineages (tunicates, insects, and nematode) in which we could detect no Bicore homologies. Our search of these lineages was extensive and included genome assemblies for 3 species of tunicate (∼0.4 Gb) and trace data for 4 species (∼7.4 Gb), genome assemblies for 21 species of insect (∼6.0 Gb) and trace data for 45 species (∼81.2 Gb), and genome assemblies for 11 species of nematode (∼1.4 Gb) and trace data for 21 species (∼15.7 Gb). Thus, it is possible that Bicores have been lost in the tunicate, insect, and nematode lineages. Corroborating this possibility, genomics studies have shown that these three lineages are perhaps the most diverged among bilaterians [46]–[48].

Even within the protostome genomes that have clear conservation of Bicores, these homologies lie at the cusp of what current computational tools can detect. For example, using human Bicore1 as a query, we can detect Bicore1 deuterostome orthologs in amphioxus, sea urchin, and acorn worm. However, we miss the critical protostome owl limpet and sea hare elements. Using zebrafish Bicore1 as the query, we detect the owl limpet element but miss the sea hare. Using amphioxus Bicore1, we detect the sea hare element but miss the owl limpet (Figure 3K). We see similar results for Bicore2 (Figure 4J). When one considers the alignments of Figure 3A and Figure 4A, it is easy to imagine how variations in spacing between co-linearly conserved binding sites may drop the overall sequence conservation below 60% identity and below the detection ability of our current tools.

While the full-length Bicores are not identical (or ultraconserved) even between human and rodents, the binding sites they encode have resisted substitutions, insertions, deletions, and rearrangements for over 600 million years in highly diverged deuterostome and protostome species. At least two fundamental questions are raised by these observations: First, have Bicores conserved their ancestral sequence while being independently co-opted in different lineages to serve unrelated contexts, or do these conserved sequences also conserve a common ancestral function (e.g. in early lineage differentiation) yet to be revealed? The second closely related question is what makes Bicores unique? The small number of identified Bicores implies extensive cis-regulatory rewiring. If the Bicores are indeed the only examples of cis-regulatory elements conserved between deuterostomes and protostomes, we are left asking what makes these enhancers different from others.

It is, however, currently hard to know what the true number of Bicores is. The initial discovery of the conservation of hox genes across bilateria ignited the field of “evo-devo” [49]. Now, scores of other bilaterian conserved developmental genes have been characterized. Over a decade later, the discovery of let-7 revealed the first example of a bilaterian conserved micro-RNA [50]. It is now appreciated that ∼30 other bilaterian conserved miRNAs exist [51]. Here we have presented the first examples of developmental enhancers conserved between deuterostomes and protostomes. We predict that as more genomes are sequenced, our understanding of cis-regulatory logic improves, and our screening is refined, more bilaterian conserved regulatory elements will be discovered. As our catalog of such genomic regions and our ability to experimentally probe these distant relatives grow, so will our understanding of the true extent of cis-regulatory sequence and function conservation underlying animal development.

## Materials and Methods

### Coding versus non-coding aligning bases

The base set of “coding exons” for Figure 1 was defined as the union of all coding bases in the Human (hg18) UCSC Genes track [52].

The base set of “placental mammal conserved non-coding elements (CNEs)” for Figure 1 was defined using the UCSC PhastCons placental mammal most conserved track [53]. From this set, we strictly removed regions that show any evidence for coding potential. To do so, we excluded any region annotated as an exon by UCSC knownGene [52], refSeq [54], or Ensembl [55]. We also excluded any region predicted to be exonic by Exoniphy [56]. Further, we excluded any region that aligns to a vertebrate or invertebrate mRNA from Genbank [57], an mRNA from the Mammalian Gene Collection [58], or a human spliced EST from Genbank [57]. Potential functional non-coding RNAs were removed by eliminating all non-coding genes annotated by Ensembl and UCSC [52], [55]. We excluded all miRNAs and snoRNAs found in miRBase and snoRNABase [59], [60]. Next, we removed all pseudogenes based on annotations from the Yale Pseudogene Database [61] and the Vertebrate Genome Annotation (VEGA) database [62]. For each potentially coding region we removed with any of the above filters, we also removed the 150 bases upstream and the 150 bases downstream. We added this stringency because the regions immediately flanking exons are often conserved, and we wish to focus on conservation that cannot be accounted for by coding or splicing related functions. Lastly, we required each CNE to be at least 50 base pairs in length and to align syntenically in mouse (mm9).

For each species comparison of either coding or non-coding bases, we counted aligning bases using the UCSC whole-genome alignments between hg18 and the following assemblies: mm9, monDom4, galGal3, xenTro1, danRer5, petMar1, braFlo1. This resulted in 34,116,513 base pairs of (1.18% of the human genome) “coding exons” conserved to mouse, and a conservative (non-overlapping) 34,124,419 base pairs (1.18% as well) of “placental mammal CNEs” conserved to mouse in Figure 1.

### Vertebrate CNEs

We used the UCSC phastCons placental mammal most conserved and vertebrate most conserved tracks to find regions of the human genome (hg18) that are the most conserved in comparisons of 32 placental mammals as well as across 44 vertebrates [53]. PhastCons elements combine the level of base pair conservation with depth of species conservation, and are not necessarily found in all genomes in either alignment. We then used the UCSC nets (a subset of the full alignments between any pair of species most likely to be orthologous) to require each element to align to at least two non-amniote vertebrates (xenTro2, tetNig1, fr2, oryLat2, gasAcu1, danRer5, petMar1), as well as to mouse (mm9).

We applied the same stringent non-coding filters used to generate our placental mammal CNE set (see above). As an added stringency, we also compared each region to the full RefSeq database using blastx and removed any element that hit a validated protein with any e-value. Lastly, we looked for overlap between our elements and regions of the genome that have predicted conserved RNA secondary structure [63]. Elements with ≥60% of bases overlapping such regions were removed. We required each element to be at least 50 base pairs, and in total we generated a conservative set of 8,069 vertebrate CNEs, covering 1.7 Mb (0.06%) of the human genome.

### Screen for bilaterian conserved regulatory elements

To identify elements most likely to be conserved across bilaterians, we searched for elements with the strongest signatures of conservation within deuterostomes. We compared all 8,069 of our vertebrate CNEs to the published Ciona (ci2) [15], Amphioxus (braFlo1) [16], and Sea urchin (strPur2) [17] genomes. First, we soft masked low complexity sequences. We then used lastz [14] to search each element against each genome. We ran lastz using very low thresholds (hspthresh = 1500, gappedthresh = 2500) [64]. We find these to increase sensitivity but also result in many dubious alignments. We thus further filtered the lastz hits, keeping only alignments with at least 65% identity and an entropy score of 1.7 or greater. The entropy of the alignment is calculated as -∑f_b_log(f_b_) for all bases b such that *f_b_*>0, where *f_b_* is the fraction of aligning bases of base b.

For each element with at least one hit passing these filters, we manually inspected the alignment and the surrounding genomic landscape. As a final filter, we only kept elements that have maintained synteny with the same target gene in vertebrates and the aligning invertebrates. We associated each vertebrate CNE with the two nearest genes in the human genome (hg18). For any hit to an invertebrate genome, we found the nearest annotated mRNAs and compared these to the database of validated Refseq proteins using blastx [65]. If the top hit for this search was an ortholog of the appropriate human target gene, then we called the hit syntenic (Table S4 and S5). Five vertebrate CNEs had at least one such syntenic hit (Table S2).

To more fully characterize the evolution of these five elements across the metazoan tree of life, we then performed a second more comprehensive and more sensitive search. For each of the five CNEs, we extracted all vertebrate instances of the element using the UCSC 44-way multiple alignment [66] on the hg18 genome browser. As a query to our search, we used all vertebrate instances of each element as well as the previously discovered invertebrate instances. Each query was searched against all available non-vertebrate metazoan sequence data (Figure S1).

Each new hit found with this comprehensive search was then used as a query to repeat the search process until no new hits were found. We manually inspected all hits, checking for the quality of the alignment and for gene synteny. For hits to genomes without an annotated set of mRNAs, we checked for synteny using nearby spliced ESTs (Table S4 and S5). Of the five elements, one was conserved in chordates, two were conserved in deuterostomes, and two were conserved in both deuterostomes and protostomes (Table S2).

### Other computational analysis

Multiple alignments were generated using ClustalW [67] and manually edited using JalView [68]. Conservation profiles of the multiple alignments were generated using WebLogo [69]. Conservation profiles were compared to a library of position weight matrices (PWMs) from Uniprobe [70], TRANSFAC [71], and GENOMATIX. PWMs that best match the substitution pattern in the multiple alignment were manually chosen and aligned to the conservation profile.

### Zebrafish transgenics

Human, zebrafish, sea urchin, owl limpet and tick sequences were PCR amplified from genomic DNA samples or synthesized (Genescript, Piscataway, NJ) (Dataset S1 and Table S7). All sequences were cloned into the E1B-GFP Tol2 vector [72] using the D-TOPO and Gateway cloning systems (Invitrogen, Life Technologies Corporation). Wildtype AB strain zebrafish were bred according to standard methods and 1-cell stage embryos were injected with the enhancer assay vector and Tol2 transposase mRNA according to previously described methods [73]. Embryos were examined for GFP expression at 6 hours post fertilization (hpf), 21hpf and 48hpf. A minimum of two independent transgenic experiments was done for each construct, and over 60 morphologically healthy embryos were scored for GFP expression at each time point.

To test for background patterns that may be generated by our expression vector, we injected the empty vector, lacking any added enhancer sequence. We could appreciate weak expression in about 50 percent of fish. However, expression was too minimal to determine cell-type using the dissecting microscope. Using confocal microscopy, we could identify expression in single cells. The most common cell types that showed such weak expression were notochord, skeletal muscle, and heart (Figure S4).

### Mouse transgenics

The human genomic region encompassing Bicore2 was synthesized with flanking NotI restriction sites (Genescript, Piscataway, NJ) and cloned into the Not5'hsp68lacZ minimal promoter expression vector [74]. The construct was linearized with SalI prior to injection. Transgenic mice were generated by pronuclear injections of FVB embryos (Xenogen Biosciences, Cranberry, NJ). Embryos were harvested at embryonic day 11.5, fixed, and whole mount stained for lacZ as described [74].

### Sea urchin transgenics

The sea urchin Bicore1 sequence was PCR amplified from *Strongylocentrotus purpuratus* genomic DNA. The product was digested and cloned into the EcoRI-BglII sites of the EpGFPII vector [28]. Carrier DNA was prepared using HindIII digestion of *S. purpura* sperm, followed by phenol chloroform extraction and precipitation with sodium acetate. The carrier DNA was brought to 0.5–1 ug/uL, spun, and filtered with a 0.2 uM filter. The construct DNA was mixed with carrier DNA at a 1∶3 molar ratio. 50% glycerol was added to the mixture to make the construct DNA a final concentration of 2,000 molecules/2pl. Over 1,000 fertilized eggs were injected and over 700 embryos were scored.

### Ethics

All animals were treated under protocols #18487 and #21758 approved by Stanford University Institutional Animal Use and Care Committee.

## Supporting Information

Dataset S1 DNA sequences tested in enhancer assays.
(PDF)

Figure S1 Computational screen for bilaterian conserved regulatory elements. Lastz was used to screen vertebrate CNEs for matches to the ciona (ci2), amphioxus (braFlo1), or sea urchin (strPur2) genomes. Hits passing our filters and manual curation were searched against all publicly available non-vertebrate metazoan sequence data. All hits were manually inspected.
(PDF)

Figure S2 The multiple alignment of Bicore1 shown in Figure 3A, with human and zebrafish paralogs added to the alignment.
(PDF)

Figure S3 (A) Expression pattern driven by human, zebrafish, sea urchin, and owl limpet Bicore1 sequences at 21 hours post fertilization compared to (B) the in-situ hybridization of Id1, courtesy of zfin.org.
(PDF)

Figure S4 Examples of background expression driven by our empty zebrafish vector.
(PDF)

Table S1 GREAT (http://GREAT.stanford.edu/ v1.8.2) [13] results for 8,069 vertebrate conserved non-coding elements in the human (hg18) genome. Top term by p-value and fold enrichment is shown for each ontology.
(PDF)

Table S2 Five candidate elements that were used as queries to search for bilaterian conserved regulatory elements.
(PDF)

Table S3 The 47 non-vertebrate metazoans whose genomes we searched.
(PDF)

Table S4 Distance between instances of Bicore1 and the closest annotated transcript or spliced EST and top Blastx hit when searching the transcript against the Refseq protein database. All instances are upstream (5′) of their respective transcript.
(PDF)

Table S5 Distance between instances of Bicore2 and the closest annotated transcript or spliced EST and top Blastx hit when searching the transcript against the Refseq protein database. All instances are upstream (5′) of their respective transcript.
(PDF)

Table S6 Number of embryos screened for each construct and the percent of GFP expressing embryos that exhibited the specified pattern shown in Figure 3 and Figure 4.
(PDF)

Table S7 Primers used to amplify out Bicore sequences for zebrafish transgenic enhancer assays.
(PDF)

Text S1 Brief summary of past studies that have searched for enhancers conserved between deuterostomes and protostomes.
(PDF)
