# Supplementary material for: Human Developmental Enhancers Conserved between Deuterostomes and Protostomes
Source: PLoS Genet. 2012 Aug 2;8(8):e1002852. doi: 10.1371/journal.pgen.1002852 (PMC3410860; doi:10.1371/journal.pgen.1002852)
Supplement: Dataset S1 — DNA sequences tested in enhancer assays. (PDF) [file pgen.1002852.s001.pdf]

Dataset S1.

Sequences tested in zebrafish transgenic enhancer assays

```
>Bicore1_human
CTAGCTAGACCAGTTTGTCGTCTCCATGGCGACCGCCCGCGCGGCGCCAG
CCTGACAGTCCGTCCGGGTTTTATGAATGGGTGACGTCACAGGCCTGGCG
TCTAACGGTCTGAGCCGCTGGTTCAGACGCTGACACA

>Bicore1_zebrafish
TGCCAGCCTGTTGTTTGTTGTTGCTATAGGGACCGCCCGCTCGGCGCCAG
GCTGACAGGCGTCAAGGCTCGAATGAATGGGTGACGTCACGGCGCTGGCG
CCCGGCAGTCTGCTTCGGTTTGTTTGGACAAT

>Bicore1_urchin
GTTACTATGGCGACCGCCAGACCGGCGCCCGTCTGCTATCGGATTGTGGG
TGGATGAATGGGTGACGTCACAGATCGTGGCGCCGGTCTGTCGCCGTGAG

>Bicore1_limpet
TATCAGTGAGTCGAGGGCGGCTTCTTTGAAGTTATGTTTGATTGACAGGT
ATGTAACGCTAGCGACCGACTATACGGCGCCGGCTCGAGAGGAAATACAT
ACAGTGTTGAATGGGTGACGTCACAAGTGCGGCGCCAGTCAGTCTGCTAG
CTGAATTGTTGATTGTAGAGGTTCACATCGAGTTATCACGTGCAGAAAAC

>Bicore2_human
CTCCCACCCTGTTTTCCTCCCTCCCCCCCTTCTTTGGGCATCTCCACCCC
TCCATCAATTGTCAATGTTCCTCGACCGCAATCAATCAGTTATTTGTCAG
CTCTTGTCAATCCTCCCGTGATTTATGTCAGCTTTTGTTGCTGATTACAA
GGCGGGTGCGACTTGAAGGGAAAAAGAGAGAGGGAGAGAGAGACGGAGAG
GAAGGAGGAGATTGAGAGGGAACTGGAGGAGGGGAAAAGAGGAGCGGCCT

>Bicore2_zebrafish
AAGTGACTCTTCTTCTGGATGCTTCTTTTGCCCTTTTCTCTGTCACTCGC
GGCACATAATCAGCTTCAAAAGCTGACGTGAAGTAAGAAAAGGATTGACA
GCGACTGACAAATAACTGATTGATTGCGGTCGAGCAGAATTGACAGTTGA
CGGAGGGGTGGGGATAGAAATAGAAGGGCGGTGTATATTATATTGAAAAC
ATGTGACATTCACATCCCTCTATCACTGCATTGGTCTGCTCTTGT

>Bicore2_urchin
CGATTTATCTCGGGGAATCATACCCCCTTTCAAAAGCCGTTTTTCAAATT
ACTCGAGCCATGTTATCAACAACAAAACGCTGACATAAATCAGACGGAAT
TGACAAGGCTGACACCGCATTTGATTGATCGCAGAACAGCGAGCGGCACT
GGACGCCTGCCAACGGGAATTGCTAATCGCGAATTTGATTGGCTGGCGCG
GAGCATGTGACAGGATCACATATCAATCATCCCCG

>Bicore2_tick
CCGACGGCCGTCCACCCAAGCGGCCCAAAGGAAGACCGCCCCGGCTCTGA
CAACAAAAGCTGACATAAATCATGCCTGATTGGCAGCTCTGACAACGGGC
TGATGGATAGCGTTGTAAAAGTTGACAGCTGGCCCCACGCCAGATCATTA
GCTCGGCCGGGCTTCCATTGGCCGCCGCGACCGCGGAGCGCTTCGGGGGG
```

Sequence tested in sea urchin transgenic enhancer assay

```
>Bicore1_urchin_long
CGTGGAGGCGTCAAATAGTCTCTGCAATCTACAAGCGCAGACCTAACACA
AAAATCCGATATAAAAGACGATTGCCATCTTATGGTCTTCATTTGTATTT
TGACGGATTTTTTAGTAGAATATGAGCAGATAGTACTGATATGGTTTTGT
AGACAAATAGGATATCATAATCCTGAAAATCTGTATTTTAAACTTCCTCA
AAAGTTTTGGACTTTCTTAAAGGTCTGTAAAGTAGACTTGGCAATGTCTC
AGGCGGGAGTGCAGGTGTGCAAGCGACGGGTGTGAATAGTGGGCGCCCCC
AAAGACCCGACCACCAGTTTAGCAAAGTGACGATCCCAAAAGCCATTTAT
CGCTGCTCTCGATAGTTTGTTAGTAATTGATTCAGGCTGCGGGACGCCTT
TGATGTTGGTGATTGGCGTGTGCGTTACTATGGCGACCGCCAGACCGGCG
CCCGTCTGCTATCGGATTGTGGGTGGATGAATGGGTGACGTCACAGATCG
TGGCGCCGGTCTGTCGCCGTGAGCTCTGGTAGTATTGGAGGGGGGCTTAG
GAGGGCCAATTAATCACCTGTCACTGGGAACCAGCGGAGTTCCACCACAC
CCCTAGCCTCAAAATACGAACCCCTGTACAACAGGTGGATGCCACAGACA
ACTAACATTATCCATCCGAACATTCTGATACTCCAGATGATTAATGCAAC
CATTCCAGAAATGAAGTCGCTCTATTTTAGGGTACTTTAAAAAGTTACGA
AACAATAATAATGCAATAAGAATACCGAATGTATGGATACATTTTCGGTT
TGAGACTACGAAGTCTCGGGGGAGGTGTATGAGCTTATGAGGGGTATGTT
TACAGGTTTACCTATGGTAAAGAATAGGGTGAGTGTTTGGATTACCATCA
TGTTCGTCTGCACAGCTGTGAACTGGTTCATGTGACCGCCATATACCAAG
G
```

Sequence tested in mouse transgenic enhancer assay

```
>Bicore2_human
CCAGAAGGAGCACCGGTGGGAGTGTGGACACCGGCCGGACTGTCAACTCCAGGGGCGAAGGGAACCTGCACACCCAG
TGTTTTTTCCTTCGCAGTAATCGAGATCCCGCGCGGCGCAGCGCAGCCACCAGGGTAAGAAGGCAAGGTGGGGAGCC
GGAGCTGGAAGAAGCCCGCCCGCCCGCTCTAATTTCCTCAGATTCCGCGGCGGAGAAACCAGAAGCTAGATGGGCAG
TCGCAGCGGCGGCGGCTCAACACCGCGAGGAGCGCTGGGCTCTCCGCCCTTCCCGGCCACGTGACGCCCGGGGACGC
GTAGATTGGGGCAGCAGCGGGGGTCACATGTTTCCTCTGTTTCACCCTCAGTCTGTCCCCCAACCCCCCATTCTTAC
TCTCCCACCCTGTTTTCCTCCCTCCCCCCCTTCTTTGGGCATCTCCACCCCTCCATCAATTGTCAATGTTCCTCGAC
CGCAATCAATCAGTTATTTGTCAGCTCTTGTCAATCCTCCCGTGATTTATGTCAGCTTTTGTTGCTGATTACAAGGC
GGGTGCGACTTGAAGGGAAAAAGAGAGAGGGAGAGAGAGACGGAGAGGAAGGAGGAGATTGAGAGGGAACTGGAGGA
GGGGAAAAGAGGAGCGGCCTCCTGGGATGGGGGTGGGGTGGGGGCTCTAAGAAAAAGAATGAAAGAGGCGCACGGTG
TCAGGAAAATGAATAGCGAGAGTAAAGTGCGCAGGTGCGCCCAGGGCGCCGAGAGGGGCGCGCAGGCCTGGAGTGTG
CGCCTGCCCTCTCGGTGTCGGAGAGACGCCCTTCCACCTCTGGGAGCCTCGGTCTGTTGGGGTCGCGGAGTTCGGGC
GCGGCTCCGGGTACCCGAGACCAGCGGCGGCAACTTCTAACACGGGAGATTTCCCGCCACCCCACCCCGCCGCCGCG
AGTCCTCGCGGGGCGTGTTGCGTGCGGAGGTCAGGCTGCCACCCTCTGTAGTTCCCTAACCCCAAACTCGGAGACT
```
