# Supplementary figures and images for: Human Developmental Enhancers Conserved between Deuterostomes and Protostomes
Source: PLoS Genet. 2012 Aug 2;8(8):e1002852. doi: 10.1371/journal.pgen.1002852 (PMC3410860; doi:10.1371/journal.pgen.1002852)

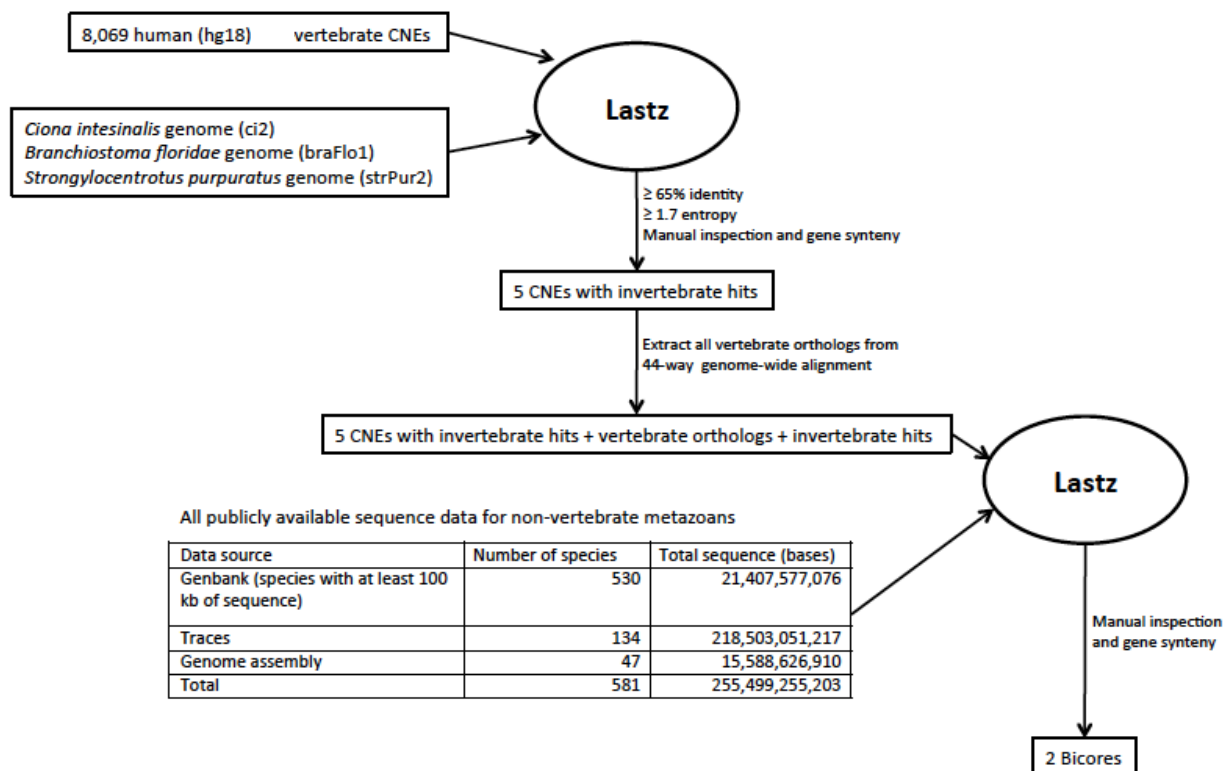

**Figure S1.**

Supplement: Figure S1 — Computational screen for bilaterian conserved regulatory elements. Lastz was used to screen vertebrate CNEs for matches to the ciona (ci2), amphioxus (braFlo1), or sea urchin (strPur2) genomes. Hits passing our filters and manual curation were searched against all publicly available non-vertebrate metazoan sequence data. All hits were manually inspected. (PDF) [file pgen.1002852.s002.pdf]

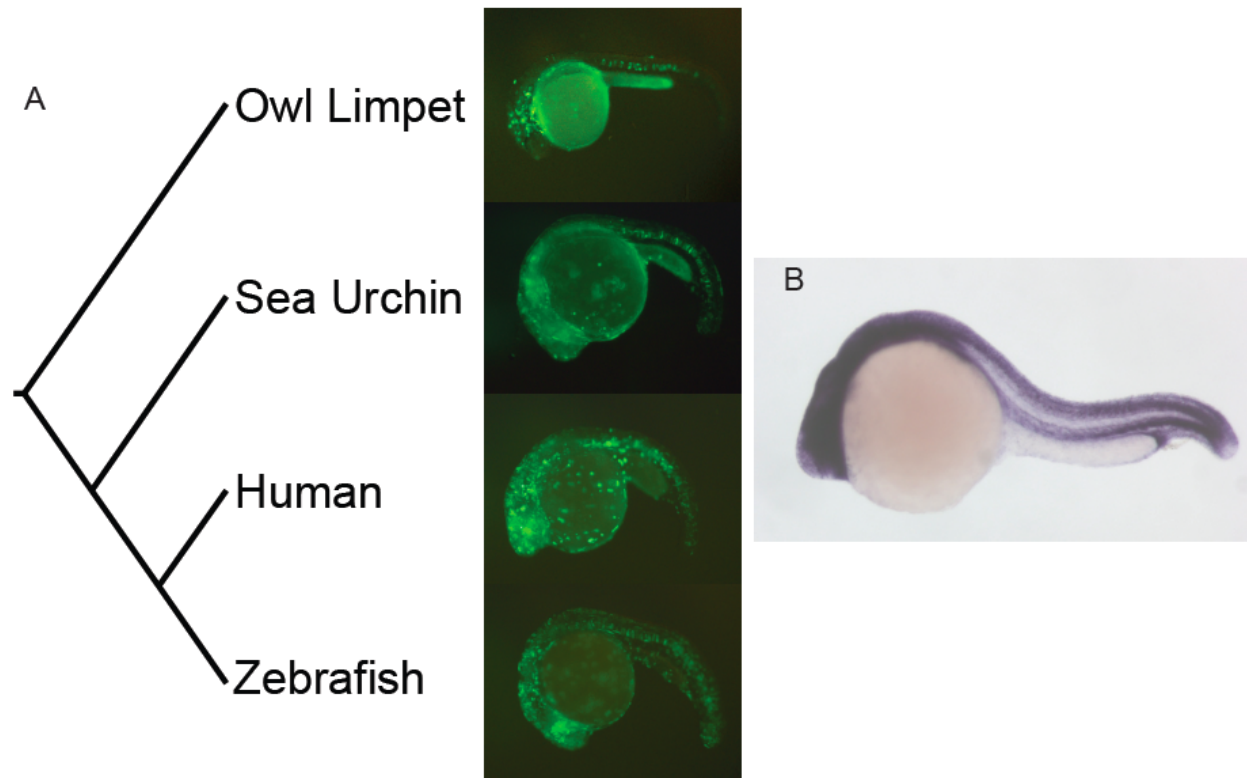

**Figure S3.**

Supplement: Figure S3 — (A) Expression pattern driven by human, zebrafish, sea urchin, and owl limpet Bicore1 sequences at 21 hours post fertilization compared to (B) the in-situ hybridization of Id1, courtesy of zfin.org. (PDF) [file pgen.1002852.s004.pdf]

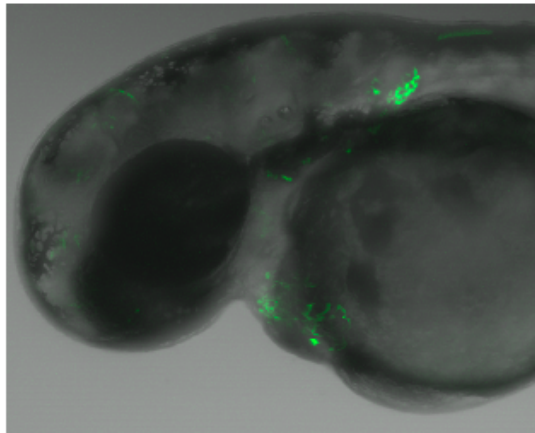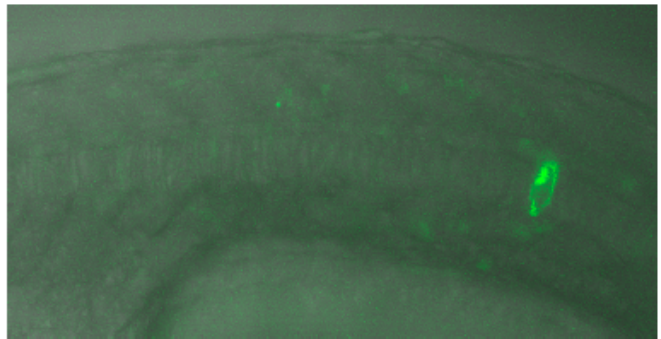

**Figure S4.**

Supplement: Figure S4 — Examples of background expression driven by our empty zebrafish vector. (PDF) [file pgen.1002852.s005.pdf]
