# Supplementary material for: Human Developmental Enhancers Conserved between Deuterostomes and Protostomes
Source: PLoS Genet. 2012 Aug 2;8(8):e1002852. doi: 10.1371/journal.pgen.1002852 (PMC3410860; doi:10.1371/journal.pgen.1002852)
Supplement: Figure S2 — The multiple alignment of Bicore1 shown in Figure 3A, with human and zebrafish paralogs added to the alignment. (PDF) [file pgen.1002852.s003.pdf]

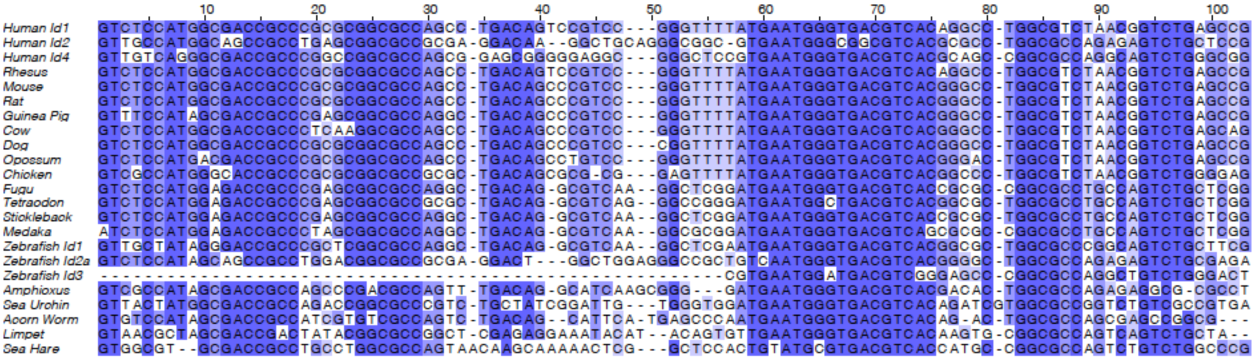
10
20
30
40
50
60
70
80
90
100
Human Id1
Human Id2
Human Id4
Rhesus
Mouse
Rat
Guinea Pig
Cow
Dog
Opossum
Chicken
Fugu
Tetraodon
Stickleback
Medaka
Zebrafish Id1
Zebrafish Id2a
Zebrafish Id3
Amphioxus
Sea Urchin
Acorn Worm
Limpet
Sea Hare

Figure S2.
