# Supplementary material for: Human Developmental Enhancers Conserved between Deuterostomes and Protostomes
Source: PLoS Genet. 2012 Aug 2;8(8):e1002852. doi: 10.1371/journal.pgen.1002852 (PMC3410860; doi:10.1371/journal.pgen.1002852)
Supplement: Table S1 — GREAT (http://GREAT.stanford.edu/ v1.8.2) [13] results for 8,069 vertebrate conserved non-coding elements in the human (hg18) genome. Top term by p-value and fold enrichment is shown for each ontology. (PDF) [file pgen.1002852.s006.pdf]

Ontology	Top term	Bonferroni corrected Binomial P-value	Fold Enrichment	Number of elements	Number of genes
GO: Molecular function	Sequence-specific DNA binding	<1.0e-324	4.4	3,157	293
GO: Biological process	Forebrain development	<1.0e-324	4.1	1,159	81
GO: Cellular component	Transcription factor complex	2.2e-183	3.6	734	65
MGI: Phenotype	Neonatal lethality	<1.0e-324	2.8	2,530	246

Table S1.
