# Supplementary material for: Human Developmental Enhancers Conserved between Deuterostomes and Protostomes
Source: PLoS Genet. 2012 Aug 2;8(8):e1002852. doi: 10.1371/journal.pgen.1002852 (PMC3410860; doi:10.1371/journal.pgen.1002852)
Supplement: Table S2 — Five candidate elements that were used as queries to search for bilaterian conserved regulatory elements. (PDF) [file pgen.1002852.s007.pdf]

**Element (hg18 coords)**	**Target gene**	**Observed depth of conservation**	**Invertebrate hits where gene synteny can be shown**
chr10:131574504-131574704	EBF3	Chordate	Amphioxus
chr8:37652008-37652247	ZNF703	Deuterostome	Amphioxus, Acorn Worm
chr4:4909505-4909664	MSX1	Deuterostome	Amphioxus, Sea urchin
BICORE1<br>chr20:29655765-29655862	ID1	Bilaterian	Amphioxus, Sea urchin, Acorn worm, Owl limpet, Sea hare
BICORE2<br>chr10:76832334-76832433	ZNF503	Bilaterian	Amphioxus, Sea urchin, Acorn worm, Tick

Table S2.
