# Supplementary material for: Human Developmental Enhancers Conserved between Deuterostomes and Protostomes
Source: PLoS Genet. 2012 Aug 2;8(8):e1002852. doi: 10.1371/journal.pgen.1002852 (PMC3410860; doi:10.1371/journal.pgen.1002852)
Supplement: Table S3 — The 47 non-vertebrate metazoans whose genomes we searched. (PDF) [file pgen.1002852.s008.pdf]

Species	NCBI Taxonomic ID
acyrthosiphon pisum	7029
aedes aegypti	7159
anopheles gambiae	7165
apis mellifera	7460
aplysia californica	6500
branchiostoma floridae	7739
caenorhabditis brenneri	135651
caenorhabditis briggsae	6238
caenorhabditis elegans	6239
caenorhabditis japonica	281687
caenorhabditis remanei	31234
capitella sp. 1	73382
ciona intestinalis	7719
ciona savignyi	51511
culex quinquefasciatus	7176
daphnia pulex	6669
drosophila ananassae	7217
drosophila erecta	7220
drosophila grimshawi	7222
drosophila melanogaster	7227
drosophila mojavensis	7230
drosophila persimilis	7234
drosophila pseudoobscura	7237
drosophila sechellia	7238
drosophila simulans	7240
drosophila virilis	7244
drosophila yakuba	7245
echinococcus multilocularis	6211
globodera pallida	36090
haemonchus contortus	6289
ixodes scapularis	6945
lottia gigantea	225164
mayetiola destructor	39758
nasonia vitripennis	7425
nematostella vectensis	45351
nippostrongylus brasiliensis	27835
oikopleura dioica	34765
pediculus humanus corporis	121224
pristionchus pacificus	54126
rhodnius prolixus	13249
saccoglossus kowalevskii	10224
schistosoma mansoni	6183
schmidtea mediterranea	79327
strongylocentrotus purpuratus	7668
strongyloides ratti	34506
tribolium castaneum	7070
trichinella spiralis	6334

Table S3.
