# Supplementary material for: Human Developmental Enhancers Conserved between Deuterostomes and Protostomes
Source: PLoS Genet. 2012 Aug 2;8(8):e1002852. doi: 10.1371/journal.pgen.1002852 (PMC3410860; doi:10.1371/journal.pgen.1002852)
Supplement: Table S4 — Distance between instances of Bicore1 and the closest annotated transcript or spliced EST and top Blastx hit when searching the transcript against the Refseq protein database. All instances are upstream (5′) of their respective transcript. (PDF) [file pgen.1002852.s009.pdf]

Gene	Distance to Bicore1 (Kb)	Transcript Identifier	Top Protein Blastx Hit (NP only)	E-value
Human ID1	0.9	Refseq: NM_002165	NP_001091037.1 (Cow Id1)	5.0E-63
Human ID2	3.0	Refseq: NM_002166	NP_034626.1 (Mouse Id2)	4.0E-68
Human ID4	32.4	Refseq: NM_001546	NP_112443.1 (Mouse Id4)	4.0E-47
Zebrafish Id1	1.6	Refseq: NM_131245	NP_001187857.1 (Catfish Id1)	2.0E-53
Zebrafish Id2a	1.6	Refseq: NM_201291	NP_001118195.1 (Rainbow trout id2)	2.0E-60
Zebrafish Id3	5.7	Refseq: NM_152967	NP_989920.1 (Chicken Id3)	1.0E-34
Amphioxus Id	0.6	Refseq: XM_002609573	NP_988885.1 (Xenopus Id2)	2.0E-15
Sea urchin Id	3.1	Refseq: XM_775662	NP_001161570.1 (Acorn worm Id)	3.0E-12
Acorn worm Id	0.5	Refseq: NM_001168098	NP_112443.1 (Mouse Id4)	1.0E-17
Owl impet Id	0.3	JGI Gene Catalog Transcrip Id: 233168	NP_001165446.1 (Xenopus Id4)	4.0E-09
Sea hare Id	0.9	Spliced Est: FF075156	NP_001134882.1 (Salmon Id2)	1.0E-03

Table S4.
