# Supplementary material for: Human Developmental Enhancers Conserved between Deuterostomes and Protostomes
Source: PLoS Genet. 2012 Aug 2;8(8):e1002852. doi: 10.1371/journal.pgen.1002852 (PMC3410860; doi:10.1371/journal.pgen.1002852)
Supplement: Table S5 — Distance between instances of Bicore2 and the closest annotated transcript or spliced EST and top Blastx hit when searching the transcript against the Refseq protein database. All instances are upstream (5′) of their respective transcript. (PDF) [file pgen.1002852.s010.pdf]

Gene	Distance to Bicore2 (Kb)	Transcript Identifier	Top Blastx Hit (NP only)	E-value
Human ZNF503	0.8	Refseq: NM_032772	NP_663434.2 (Mouse Zfp503)	0.0
Zebrafish Znf503	0.7	Refseq: NM_198840	NP_001133583.1 (Salmon Znf503)	0.0
Amphioxus Znf503	0.3	Refseq: XM_002596090	NP_001161611.1 (Acorn worm NocA-like transcription factor)	7.0E-24
Sea urchin Znf503	0.4	Refseq: XM_775815	NP_001161611.1 (Acorn worm NocA-like transcription factor)	1.0E-64
Acorn worm NocA-like transcription factor (Znf503)	0.3	Refseq: NM_001168139	NP_942137.1 (Zebrafish Znf503)	5.0E-42
Tick Noc (Znf503)	0.4	Vectorbase: ISCW005133	NP_476917.1 (Drosophila Noc)	2.0E-38

Table S5.
