# Supplementary material for: Human Developmental Enhancers Conserved between Deuterostomes and Protostomes
Source: PLoS Genet. 2012 Aug 2;8(8):e1002852. doi: 10.1371/journal.pgen.1002852 (PMC3410860; doi:10.1371/journal.pgen.1002852)
Supplement: Table S6 — Number of embryos screened for each construct and the percent of GFP expressing embryos that exhibited the specified pattern shown in Figure 3 and Figure 4. (PDF) [file pgen.1002852.s011.pdf]

Element	Embryos screened	GFP expressing embryos	Specified pattern	%GFP with specified pattern
Human Bicore1	64	35	26	74.3
Zebrafish Bicore1	80	67	45	67.2
Sea urchin Bicore1	88	63	31	49.2
Owl limpet Bicore1	112	77	68	88.3
Human Bicore2	71	51	51	100.0
Zebrafish Bicore2	71	17	13	76.5
Sea urchin Bicore2	69	41	32	78.0
Tick Bicore2	65	32	29	90.6

Table S6.
