# Supplementary material for: Human Developmental Enhancers Conserved between Deuterostomes and Protostomes
Source: PLoS Genet. 2012 Aug 2;8(8):e1002852. doi: 10.1371/journal.pgen.1002852 (PMC3410860; doi:10.1371/journal.pgen.1002852)
Supplement: Table S7 — Primers used to amplify out Bicore sequences for zebrafish transgenic enhancer assays. (PDF) [file pgen.1002852.s012.pdf]

Locus	Primer1	Primer2
Human Bicore1	CAGTTTGTCGTCTCCATGGCGACC	TCTGAACCAGCGGCTCAGACCGT
Zebrafish Bicore1	TTGTTTGTTGTTGCTATAGGGACCGCC	CAAACCGAAGCAGACTGCCGGG
Sea urchin Bicore1	GTTACTATGGCGACCGCCAG	CTCACGGCGACAGACCGGCG
Human Bicore2	CTCCCACCCTGTTTTCCTC	AGGCCGCTCCTCTTTTCC
Zebrafish Bicore2	ACAAGAGCAGACCAATGCAG	AAGTGACTCTTCTTCTGGATGCTT
Sea urchin Bicore2	CGATTTATCTCGGGGAATCA	CGGGGATGATTGATATGTGA

Table S7.
