# Supplementary material for: Human Developmental Enhancers Conserved between Deuterostomes and Protostomes
Source: PLoS Genet. 2012 Aug 2;8(8):e1002852. doi: 10.1371/journal.pgen.1002852 (PMC3410860; doi:10.1371/journal.pgen.1002852)
Supplement: Text S1 — Brief summary of past studies that have searched for enhancers conserved between deuterostomes and protostomes. (PDF) [file pgen.1002852.s013.pdf]

Human developmental enhancers conserved between deuterostomes and protostomes

Shoa L. Clarke[1], Julia E. VanderMeer[2,3], Aaron M. Wenger[4], Bruce T. Schaar[5], Nadav Ahituv[2,3], Gill Bejerano[4,5]

[1]Department of Genetics, Stanford University, Stanford, California, USA; [2]Department of Bioengineering and Therapeutic Sciences, University of California San Francisco, San Francisco, California, USA; [3]Institute for Human Genetics, University of California San Francisco, San Francisco, California, USA; [4]Department of Computer Science, Stanford University, Stanford, California, USA; [5]Department of Developmental Biology, Stanford University, Stanford, California, USA

Corresponding author: bejerano@stanford.edu (G.B)

Text S1

Past studies

Several previous studies have searched for cis-regulatory elements conserved between deuterostome and protostome species [1–8]. To our knowledge, the earliest such study describes the HB1 element in the intron of Hoxa7 [1]. HB1 is a short 36 base pair sequence consisting of three homeodomain binding sites. The sequence is conserved among mammals but not well conserved in other vertebrates. Haerry and Ghering were able to align HB1 to *Drosophila melanogaster*, *D. funebris*, and *D. virilis* sequence [1,2]. However, only 18-20 of the 36 bases (~55%) match between mouse and fly. The longest contiguous match is 5 bases. While HB1 is found in the intron of Hoxa7, it is absent from the intron of Ubx, the drosophila ortholog to Hoxa7. Instead, it is found in the 5' UTR of Ubx [1]. Moreover, several other matches to HB1 were found in drosophila, including matches around non-hox genes [2]. Mammalian and drosophila HB1 elements are unlikely to be truly orthologous sequences. Rather, HB1 elements represent a common mechanism for the regulation of genes by homeobox transcription factors.

Similarly, Kuntz et al. has found short (<30 base pairs) sequences around hox genes that are alignable between mammals and nematode worms [3]. These sequences consist of

only 13-14 matching bases, nearly all of which are A or T. These sequences likely represent two closely spaced homeodomain binding sites, and Kuntz et al. finds numerous instances of these sequences across the Hoxa locus. Many of these non-orthologous matches may be noise, as we have found short AT rich sequences to align at high frequency across the genome.

Others have taken whole-genome approaches to searching for deuterostome-protostome conserved cis-regulatory sequences [4–8]. As we have done here, these studies began by defining conserved non-coding elements (CNEs) in the human genome. However, these previous works focused on much smaller sets of CNEs, the largest being ~1,400 human-fugu conserved elements [6]. Moreover, these studies were limited by the availability of protostome genomes and searched only genomes from drosophila species or nematode worms[4–7].

A more recent study also searched the genomes of *Capitella teleta*, *Daphnia pulex*, and *Lottia gigantea* but discovered no elements conserved between deuterostomes and protostomes. This study did, however, find an enhancer called Sox21-CNR that is conserved between deuterostomes and the cnidarian sea anemone [8]. The Sox21-CNR element was excluded from our conservative set of vertebrate CNEs because it lies immediately adjacent to a region that appears to be evolving like an exon, and overlaps two sequences with conserved predicted RNA secondary structure. To more thoroughly search for evidence of Sox21-CNR in protostomes, we subjected the element to the same computational analysis we performed for the Bicores. We searched each deuterostome instance and the sea anemone instance of Sox21-CNR against our database of non-vertebrate metazoan sequence data (Supplemental Figure 1). We found no syntenic hits to any protostome species.

References

1. Haerry TE, Gehring WJ (1996) Intron of the mouse Hoxa-7 gene contains conserved homeodomain binding sites that can function as an enhancer element in Drosophila. Proc Natl Acad Sci USA 93: 13884–13889.

2. Haerry TE, Gehring WJ (1997) A conserved cluster of homeodomain binding sites in the mouse Hoxa-4 intron functions in Drosophila embryos as an enhancer that is directly regulated by Ultrabithorax. Dev Biol 186: 1–15. doi:10.1006/dbio.1997.8582.

3. Kuntz SG, Schwarz EM, DeModena JA, De Buysscher T, Trout D, et al. (2008) Multigenome DNA sequence conservation identifies Hox cis-regulatory elements. Genome Res 18: 1955–1968. doi:10.1101/gr.085472.108.

4. Bejerano G, Pheasant M, Makunin I, Stephen S, Kent WJ, et al. (2004) Ultraconserved elements in the human genome. Science 304: 1321–1325. doi:10.1126/science.1098119.

5. International Chicken Genome Sequencing Consortium (2004) Sequence and comparative analysis of the chicken genome provide unique perspectives on vertebrate evolution. Nature 432: 695–716. doi:10.1038/nature03154.

6. Woolfe A, Goodson M, Goode DK, Snell P, McEwen GK, et al. (2005) Highly conserved non-coding sequences are associated with vertebrate development. PLoS Biol 3: e7. doi:10.1371/journal.pbio.0030007.

7. Vavouri T, Walter K, Gilks WR, Lehner B, Elgar G (2007) Parallel evolution of conserved non-coding elements that target a common set of developmental regulatory genes from worms to humans. Genome Biol 8: R15. doi:10.1186/gb-2007-8-2-r15.

8. Royo JL, Maeso I, Irimia M, Gao F, Peter IS, et al. (2011) Transphyletic conservation of developmental regulatory state in animal evolution. Proc Natl Acad Sci USA 108: 14186–14191. doi:10.1073/pnas.1109037108.
